# Experimental Andes Virus Infection in Deer Mice: Characteristics of Infection and Clearance in a Heterologous Rodent Host

**DOI:** 10.1371/journal.pone.0055310

**Published:** 2013-01-31

**Authors:** Jessica R. Spengler, Elaine Haddock, Don Gardner, Brian Hjelle, Heinz Feldmann, Joseph Prescott

**Affiliations:** 1 Laboratory of Virology, Division of Intramural Research, National Institute of Allergy and Infectious Disease, National Institutes of Health, Rocky Mountain Laboratories, Hamilton, Montana, United States of America; 2 School of Veterinary Medicine, University of California Davis, Davis, California, United States of America; 3 Rocky Mountain Veterinary Branch, Division of Intramural Research, National Institute of Allergy and Infectious Diseases, National Institutes of Health, Rocky Mountain Laboratories, Hamilton, Montana, United States of America; 4 Departments of Pathology, Biology and Molecular Genetics and Microbiology, University of New Mexico HSC, Albuquerque, New Mexico, United States of America; Public Health Agency of Canada, Canada

## Abstract

New World hantaviruses can cause hantavirus cardiopulmonary syndrome with high mortality in humans. Distinct virus species are hosted by specific rodent reservoirs, which also serve as the vectors. Although regional spillover has been documented, it is unknown whether rodent reservoirs are competent for infection by hantaviruses that are geographically separated, and known to have related, but distinct rodent reservoir hosts. We show that Andes virus (ANDV) of South America, carried by the long tailed pygmy rice rat (*Oligoryzomys longicaudatus*), infects and replicates *in vitro* and *in vivo* in the deer mouse (*Peromyscus maniculatus*), the reservoir host of Sin Nombre virus (SNV), found in North America. In experimentally infected deer mice, viral RNA was detected in the blood, lung, heart and spleen, but virus was cleared by 56 days post inoculation (dpi). All of the inoculated deer mice mounted a humoral immune response by 14 dpi, and produced measurable amounts of neutralizing antibodies by 21 dpi. An up-regulation of *Ccl3*, *Ccl4*, *Ccl5*, and *Tgfb*, a strong CD4^+^ T-cell response, and down-regulation of *Il17*, *Il21* and *Il23* occurred during infection. Infection was transient with an absence of clinical signs or histopathological changes. This is the first evidence that ANDV asymptomatically infects, and is immunogenic in deer mice, a non-natural host species of ANDV. Comparing the immune response in this model to that of the immune response in the natural hosts upon infection with their co-adapted hantaviruses may help clarify the mechanisms governing persistent infection in the natural hosts of hantaviruses.

## Introduction

Pathogenic New World hantaviruses (Family: *Bunyaviridae*) are rodent-borne negative-sense RNA viruses that were first described in 1993 in the Four Corners region of the United States following an outbreak of an acute respiratory distress syndrome associated with high human mortality [1]. Worldwide, hantaviruses cause two distinct diseases; hemorrhagic fever with renal syndrome and hantavirus cardiopulmonary syndrome (HCPS, or HPS), caused by Old World and New World viruses, respectively. Several hantavirus species are highly pathogenic and infection results in high case fatality rates in humans [2]. Currently, there are no approved vaccines for New World hantaviruses [3], [4], and treatment is largely supportive. Since their discovery in North America, pathogenic hantaviruses have been identified as causing HCPS in Central and South America, where infection can also result in high mortality [5]. Sin Nombre virus (SNV) and Andes virus (ANDV) are the principal etiologic agents of HCPS in North America and South America, respectively.

Unlike other bunyaviruses, members of the genus *Hantavirus* are not transmitted by an arthropod vector, but instead are transmitted directly from rodent hosts to humans by inhalation of dried excreta, and thus, these animals serve as the vectors and reservoirs of these viruses [6], [7], [8], [9], [10]. Each hantavirus is associated with a distinct rodent host species [11], and there is a congruence between the rodent and hantavirus phylogenetics [12]. Thus, the closely related SNV and ANDV are associated in nature with hosts that are themselves closely related. The relationship between hantaviruses and their respective rodent or insectivore hosts [11] is likely a reflection of past geographic isolation and has been considered to be the result of co-adaptation with specific divergent host species. The frequent occurrence of host-switching in hantavirus evolution, however, has recently led this model to become the subject of controversy [13]. Thus, the mechanisms by which hantaviruses might go from a spillover infection of a sympatric animal species to become capable of successfully adapting to that species are almost completely unexplored experimentally. Spillover events in nature are limited, but occasionally occur within a geographic region and have been documented for several viruses and rodent host species [11], [14], providing further evidence that heterologous host species are susceptible to infection. In contrast to experimental infections, it is difficult to determine whether the presence of the genome of a hantavirus in non-reservoir host is the result of a persistent or transient infection.

HCPS is thought to be, in part, a disease of immune dysregulation [15], [16], [17]. Of the known HCPS-causing hantavirus-reservoir species relationships, SNV infection in deer mice has been most extensively investigated both experimentally and in the field. In deer mice experimentally infected with SNV, CD4^+^ T cells responses to viral N antigen occur, but are relatively weak in both acutely and persistently infected animals. However, the specific cytokine profile differed between acutely and persistently infected animals [18], suggesting a possible role in immune permissiveness to persistence by the development of regulatory T cell (Treg) responses. Characterization of the immune response in rodent reservoirs inoculated with a heterologous hantavirus might lend insight into how the immune response allows for persistence, or facilitates clearance of the virus, which, in turn, can lead to insights about the steps required for adaptation of a hantavirus to a new reservoir species.

Experimental hantavirus inoculations of a heterologous rodent species that serves as reservoir for another species of hantavirus have not been performed. SNV and ANDV are the most important HCPS-causing agents in the Americas, and it is unknown whether the rodent hosts of these viruses respond in a similar way immunologically to limit pathology in the hosts, and whether infection might result in persistent infection. Inoculation of a heterologous host with a hantavirus has at least four potential outcomes; *i*. a persistent infection without disease, as occurs in natural reservoir hosts of hantaviruses, *ii*. virus-induced pathology and disease, as occurs in humans, *iii*. clearance of the virus by the immune response without disease, or *iv*. an inability of the virus to replicate and establish an infection. In this study, we sought to examine the course of infection and immune responses of deer mice inoculated with ANDV, with the purpose of correlating the activities of the immune response with the outcome of infection. To do this, we chose to utilize the deer mouse, as it is the most extensively studied host of New World hantaviruses and a modest number of regents are available for examining the immune responses in this species. We found that ANDV transiently infects deer mice, without causing disease. This is in stark contrast to deer mice infected with SNV, which remain persistently infected for life, as well as humans, who develop HCPS [19], [20]. Deer mice respond to ANDV immunologically by up-regulation of several genes involved in the immune response, and by the production of neutralizing antibodies.

## Materials and Methods

### Biosafety and ethical statement

All work with ANDV-infected deer mice and potentially infectious materials was conducted in the biosafety level 4 (BSL4) facility at the Rocky Mountain Laboratories (RML), Division of Intramural Research, National Institute of Allergy and Infectious Diseases, National Institutes of Health. Sample inactivation and removal from the BSL4 was performed according to standard operating protocols approved by the local Institutional Biosafety Committee. All animal experiments were approved by the Institutional Animal Care and Use Committee of the Rocky Mountain Laboratories and performed following the guidelines of the Association for Assessment and Accreditation of Laboratory Animal Care, International (AAALAC) by certified staff in an AAALAC-approved facility.

### Animal infections and virus

We obtained deer mice (*Peromyscus maniculatus rufinus*) from the University of New Mexico “Manzano” colony, which were bred and maintained at RML [21]. This colony was originally generated from New Mexican deer mice and has been extensively used to study interactions with SNV. Six- to 12-week old male deer mice were anesthetized (3–5% isoflurane in 5 liters/min 100% oxygen) and inoculated with 200 ffu of ANDV-9717869 intramuscularly (i.m.) in the hind limbs (2×50 µL). One, 7, 14, 21, and 56 dpi, groups of 6 deer mice were euthanized and blood and tissues collected for processing. ANDV-9717869 was originally isolated from a pygmy rice rat (*O. longicaudatus*) captured in Chile in 1997 [22] and was propagated and titrated by immunofocus assay on Vero E6 cells.

### Isolation, culture and infection of deer mouse embryonic fibroblasts (PMEFs)

Two 13–16 day embryos from one adult female *P. maniculatus* were harvested as previously described [23]. In brief, embryos were collected and washed with PBS. The torsos were isolated from the embryonic tissue, washed with PBS, minced, pooled and placed in 0.05% Trypsin-EDTA (Invitrogen) containing 1 µg/mL DNase I (Ambion) and incubated at 37°C for 15 min. Cells were filtered using a 100 µm nylon strainer, centrifuged (500× *g*, 5 min), resuspended in complete Dulbecco's minimal essential media (DMEM) (DMEM, 10% FBS, Non-essential amino acids, and penicillin/streptomycin), and plated in tissue culture flasks (passage 1). Cells used for infection experiments were of passage 3. PMEFs were seeded at a density of 5×10^4^ cells per chamber in 8-well Nunc Lab-Tek II Chamber Slide Systems (Sigma), or 1×10^5^ cells per well in 48-well plates. PMEFs were infected with ANDV CHI-9717869 at an MOI of 0.4 for 1 hr, virus was removed, cells were washed, and 2% FBS-DMEM media was added.

### Immunofluorescence and confocal microscopy

For immunofluorescence assays (IFA), slides were fixed with 4% paraformaldehyde (PFA), then removed from the BSL4 using standard operating procedures. Cells were then permeabilized using a solution of 1% BSA and 0.3% Triton X-100 for 15 min at room temperature, rinsed with PBS, blocked with 1% BSA for 1 hr at room temperature and stained with an anti-SNV N rabbit polyclonal antibody at 1∶1000, followed by Alexa Fluor 488 goat anti-rabbit IgG (H+L) (Life Technologies) at 1∶2000. Slides were then washed three times in PBS and overlaid with glass coverslips using Prolong Gold + DAPI mounting media (Invitrogen). Immunostained cells were visualized and quantified using a Zeiss LSM710 confocal microscope with ZEN software and a Zeiss Axio Scope with Axiovision software.

### ELISA

Sera were prepared from whole blood of deer mice infected with ANDV, or uninfected control deer mice. ELISAs were performed as previously described [24]. Recombinant ANDV N was used to coat microtiter plates (Nunc) at 2 µg/mL. To detect deer mouse antibodies, we used anti-*Peromyscus leucopus* IgG (H+L) (KPL) secondary antibodies. Endpoint titers were determined to be positive when the O.D. was greater than three standard deviations above the mean O.D. value of the corresponding dilution of negative control sera.

### Virus neutralization assay

Serum samples from deer mice 14, 21 and 56 dpi (6 animals per time point) and 2 control animals were serially diluted in 2-fold increments from 1∶10 to 1∶640 in DMEM. Approximately 200 infectious units of pseudotyped VSV-GFP or VSVΔG-ANDV-GPC-GFP was added to serum. Sera and pseudotyped virus was incubated for 1 hr at 37°C prior to being adsorbed on Vero E6 cells for 1 hr at 37°C. Cells were then washed with DMEM and incubated overnight in 100 µL/well of DMEM with 2% FBS with P/S and L-glut, then visualized and quantified by counting fluorescent cells. GFP expression in cells infected with pseudotyped VSV alone served as the negative controls to determine an appropriate value for a positive 80% focus reduction neutralization test *(*FRNT_80_). As an additional negative control, all sera were tested against VSV-GFP. Neutralization endpoint titers were determined as the highest dilution that had ≥ 80% reduction in GFP expressing cells.

### RNA isolation and qRT-PCR

For isolation of total RNA from PMEFs, supernatants were collected and placed in AVL lysis buffer (Qiagen) and cell lysates were collected using RLT solution (Qiagen). For isolation of total RNA from lungs and heart, tissue was homogenized in RLT and homogenates were clarified by centrifugation. Total RNA was extracted from samples in AVL using the QIAamp Viral RNA mini kit (Qiagen), and from samples placed in RLT using the RNeasy mini kit (Qiagen) following the manufacturers protocol.

qRT-PCR detection of viral S-segment genome in tissue was performed with RNA (200 ng) in a 1-step RT-PCR using ANDV-specific primers and a probe as previously described [24]. Viral RNA abundance was calculated by comparing the cycle threshold values to an *in vitro* transcribed S-segment RNA standard of known copy number. qRT-PCR was performed in triplicate. qRT-PCR detection of gene expression was performed by 2-step RT-PCR. Reverse transcription was performed using RNA (200 ng) along with TaqMan Reverse Transcription Reagents (Applied Biosystems). Subsequent gene quantification was performed with Power SYBR Green qRT-PCR Reagents kit (Applied Biosystems), using gene specific primers as previously described [25].

### Histopathology

Tissues were fixed in 10% neutral buffered formalin for a minimum of 7 days and removed from the BSL4 using standard operating procedures. Tissues were then placed in cassettes and processed with a Sakura VIP-5 Tissue Tek, on a 12 hr automated schedule, using a graded series of ethanol, xylene, and ParaPlast Extra. Embedded tissues were sectioned at 5 µm and dried overnight at 42°C prior to staining. Tissues sections were stained with hematoxylin and eosin (H&E). For immunohistochemistry, formalin fixed, paraffin embedded tissues cut in 5 µm sections were stained on the Discovery XT automated stainer (Ventana Medical Systems) using an anti-ANDV N antibody (Austral Biologicals), and the DAB map detection kit (Ventana Medical Systems). All tissue sections were examined on an Olympus BX51 light-microscope equipped with an Olympus DP70 camera and associated cellSens Dimension 1.4.1 software. Histopathology was assessed by a board certified veterinary pathologist.

## Results

### ANDV infects and replicates in deer mouse cells *in vitro*

**Figure 1:**
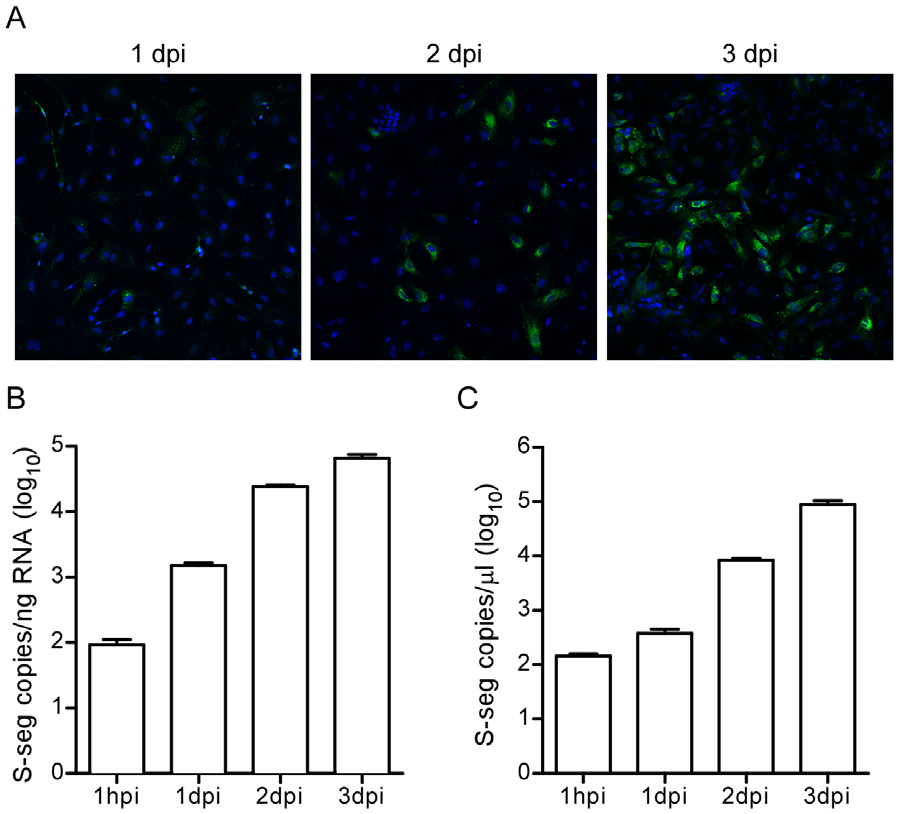
ANDV infects and replicates in PMEFs. (A) PMEFs were infected with ANDV (MOI = 0.4), fixed 1, 2, and 3 dpi, stained with an anti-N antibody, and imaged by fluorescence microscopy at 10×. The inset on 3 dpi is a 40× magnification. (B and C) Quantitative RT-PCR detection of ANDV S-segment viral RNA in PMEFs using TaqMan probes. (B) Cellular and (C) supernatant RNA was isolated at 1 h, 1, 2, and 3 dpi. Standards were serial dilutions of known copy numbers of ANDV S-segment *in vitro*-transcribed RNA, and samples were standardized by normalizing total input RNA per sample to copies/ng cell lysate RNA or copies/µL of supernatant RNA. Experiments for qRT-PCR were performed in triplicate and data represents the mean and SEM.

To determine whether deer mice might be competent for infection by ANDV, we isolated deer mouse embryonic fibroblasts (PMEFs) and inoculated them with ANDV at an MOI of 0.4 to quantitatively assess ANDV replication kinetics. Evidence of ANDV infection was seen at 1 dpi, both by immunofluorescence (Figure 1A) and qRT-PCR of ANDV S-segment (Figure 1B and C). Infection levels increased over time with an increase in the percentage of infected cells by immunofluorescence, and sequentially elevated S-segment copy numbers at both 2 and 3 dpi in both the cells and in the supernatant, suggesting a productive infection.

### Deer mice mount an antibody response to ANDV

**Figure 2:**
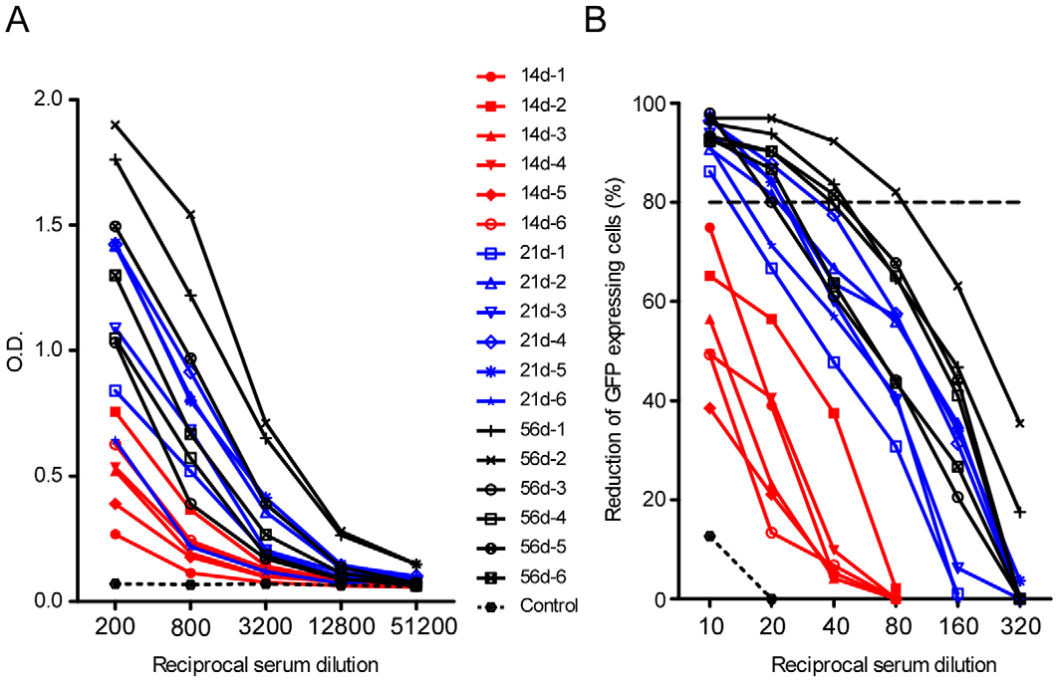
Antibody responses in deer mice inoculated with ANDV. Sera were prepared from the whole blood of ANDV-inoculated deer mice euthanized 14 (red), 21 (blue), and 56 (black) dpi, or control animals (dashed line). (A) Anti-N-specific antibody titers were determined by ELISA. (B) VSVΔG-ANDV-GPC-GFP neutralization assay. Sera from deer mice euthanized 14, 21, and 56 dpi were incubated with 200 infectious units of replication-deficient VSV pseudotyped with ANDV glycoproteins (GPC) and GFP, and assessed for neutralization ability by quantifying the reduction in GFP-positive cells. Focus reduction neutralization test (FRNT_80_) was calculated using negative control (virus alone). Neutralization titer is determined by the highest titer below the FRNT_80_. None of the serum samples tested neutralized VSV-GFP (negative control, not shown).

Specific antibody responses in deer mice inoculated with SNV are first detected as early as 10–14 dpi, but often first appear at much later time points, as late as 5 weeks post exposure [26], [27]. Deer mice were inoculated intramuscularly with ANDV to determine if they are productively infected, as this route is efficient for infecting deer mice with SNV [27]. Serum anti-N antibody levels in deer mice euthanized 14, 21, and 56 dpi were then measured by ELISA. All deer mice inoculated with ANDV developed specific antibody responses by 14 dpi, with titers of at least 800 (Figure 2A, Table 1). There was a consistent pattern of increased average antibody titers in animals euthanized at later time points post inoculation.

**Table 1:** Antibody responses and viral RNA load in deer mice inoculated with ANDV.

				Viral RNA (copies/ng RNA)^4^			
DPI^1^	Deer Mouse Designation	Anti-N Titer^2^	Neutralization titer^3^	Blood	Heart	Lung	Spleen
1	1d-1	-	-	0	1	0	0
	1d-2	-	-	0	40.95	0	0
	1d-3	-	-	0	0	0	0
	1d-4	-	-	0	0	0	0
	1d-5	-	-	0	0	0	0
	1d-6	-	-	0	0	0	0
7	7d-1	-	-	1369	0	4.1	16.11
	7d-2	-	-	0	5.6	0.5	0.15
	7d-3	-	-	4933	0.6	0	12.53
	7d-4	-	-	5125	0	0.5	3.22
	7d-5	-	-	1323	0	0.2	2.1
	7d-6	-	-	128	0	0.1	4.35
14	14d-1	800	<10	81	0	1.5	1.2
	14d-2	3200	<10	0	0	0	0
	14d-3	3200	<10	130	0	5.7	2.68
	14d-4	3200	<10	24	5.1	16.4	1.04
	14d-5	800	<10	0	0	0	0
	14d-6	3200	<10	0	166.7	1.7	0.39
21	21d-1	12800	10	0	56.1	0	0
	21d-2	12800	20	0	62.4	3	0
	21d-3	12800	20	0	281.2	0	0
	21d-4	12800	20	0	16.1	0.8	0.38
	21d-5	12800	20	0	23.2	0.2	0
	21d-6	3200	10	0	6.8	0.9	0
56	56d-1	51200	40	0	2.9	1	0
	56d-2	51200	80	0	0	0	0
	56d-3	12800	10	0	4.2	0	0
	56d-4	12800	20	0	0	0	0
	56d-5	12800	40	0	0	0	0
	56d-6	12800	20	0	0	0	0

1 Days post inoculation.

2 ELISA specific for SNV nucleocapsid antigen. ‘−’ indicates determination of titer was not performed.

3 VSVΔG-ANDV GPC-GFP FRNT_80_ neutralization test performed on Vero E6 cells.

4 Blood and organ-specific viral RNA copy number determined by TaqMan qRT-PCR.

Antibodies directed to hantavirus glycoproteins can result in virus neutralization [28], [29], [30], [31], [32], [33]. A rapid and safe neutralization assay that has previously been used, and can be performed at BSL-2, employs hantavirus glycoprotein pseudotyped vesicular stomatitis virus (VSV) [24], [34], [35]. Neutralizing antibodies in the sera of deer mice challenged with ANDV were assessed for their ability to neutralize VSV pseudotyped with the ANDV glycoprotein complex (GPC) and GFP (VSVΔG-ANDV-GPC-GFP). None of the serum samples tested neutralized VSV-GFP (negative control, data not shown). Neutralizing antibody responses did not reach the 80% focus reduction threshold in animals euthanized 14 dpi (Figure 2B, Table 1). Neutralizing titers at 21 and 56 dpi ranged from 10 to 20, and 10 to 80, respectively, with increasing average titers in groups that were tested at later time points post inoculation.

### ANDV replicates in deer mouse tissues and results in transient viremia

**Figure 3:**
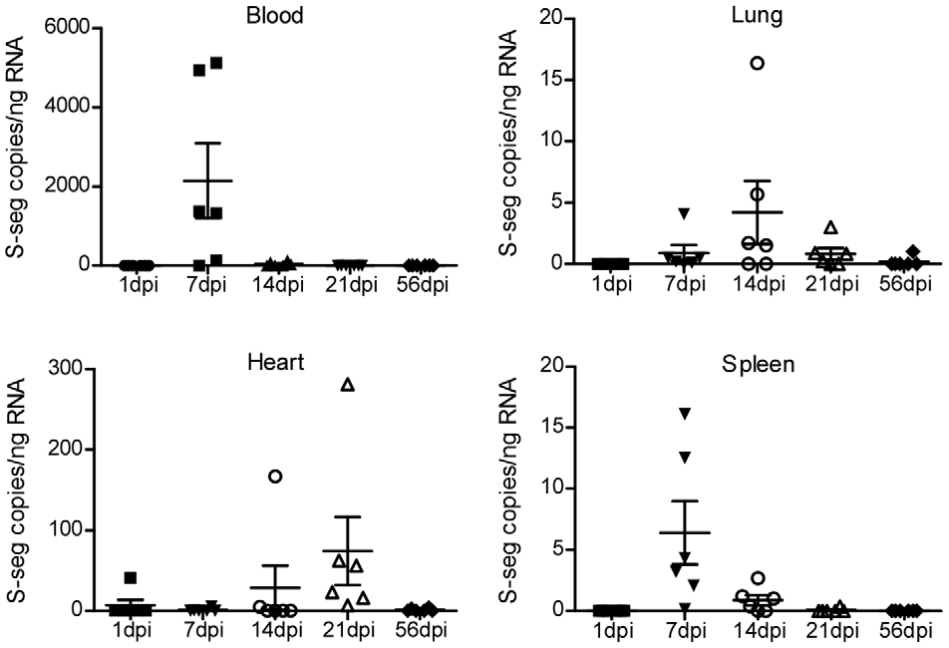
ANDV replicates in deer mouse tissues. 30 deer mice (six males per time point) were euthanized at the indicated time points following inoculation with ANDV and total RNA was extracted from the blood, lung, spleen and heart. ANDV S-segment RNA copies were measured using qRT-PCR specific for the S-segment, along with an *in vitro* transcribed RNA standard. Data was normalized to the mass of total input RNA for triplicate reactions. Error bars represent the SEM.

To examine the location of viral replication, we assessed viremia and splenic, pulmonary, and cardiac tissue-specific viral RNA levels in deer mice inoculated with ANDV at 1, 7, 14, 21, and 56 dpi by qRT-PCR analysis of ANDV S-segment RNA. A transient viremia was detected in deer mice by 7 dpi (Figure 3). Viral RNA was first detected at 1 dpi in a single heart sample, and 7 dpi in spleen and lung tissue, although at much lower levels than in the blood. Replication kinetics differed among the various tissues, with the spleen having the highest viral RNA load at 7 dpi, the lung having the highest load at 14 dpi, and the heart having the highest amount of viral RNA at 21 dpi. Overall, a large range in viral RNA load was detected within animals in each of the groups (Table 1). However, higher levels in one tissue for an individual did not correlate with higher levels in other tissues for that specific animal.

### ANDV infects deer mice without pathology

No lesions associated with ANDV infection were detected in H&E-stained tissue sections obtained from experimentally inoculated deer mice 7, 14, 21 or 56 dpi (data not shown). Additionally, no IHC staining of ANDV N protein was detected in any tissue examined at any time point (data not shown).

### ANDV infection results in the differential transcription of immune-related genes in deer mice

To investigate the immune response in deer mice inoculated with ANDV, we examined 15 immune-related genes, including those encoding subsets of chemokines, cytokines, and T cell markers, in the lungs and/or spleens at 1, 7, 14, and 21 dpi. As observed when assessing antibody responses and tissue-specific viral kinetics, a high amount of variation was found in the levels of immune markers between individual deer mice. However, even with this variation present, significant differences in immune markers were seen compared to control animals at various time points post inoculation.

**Figure 4:**
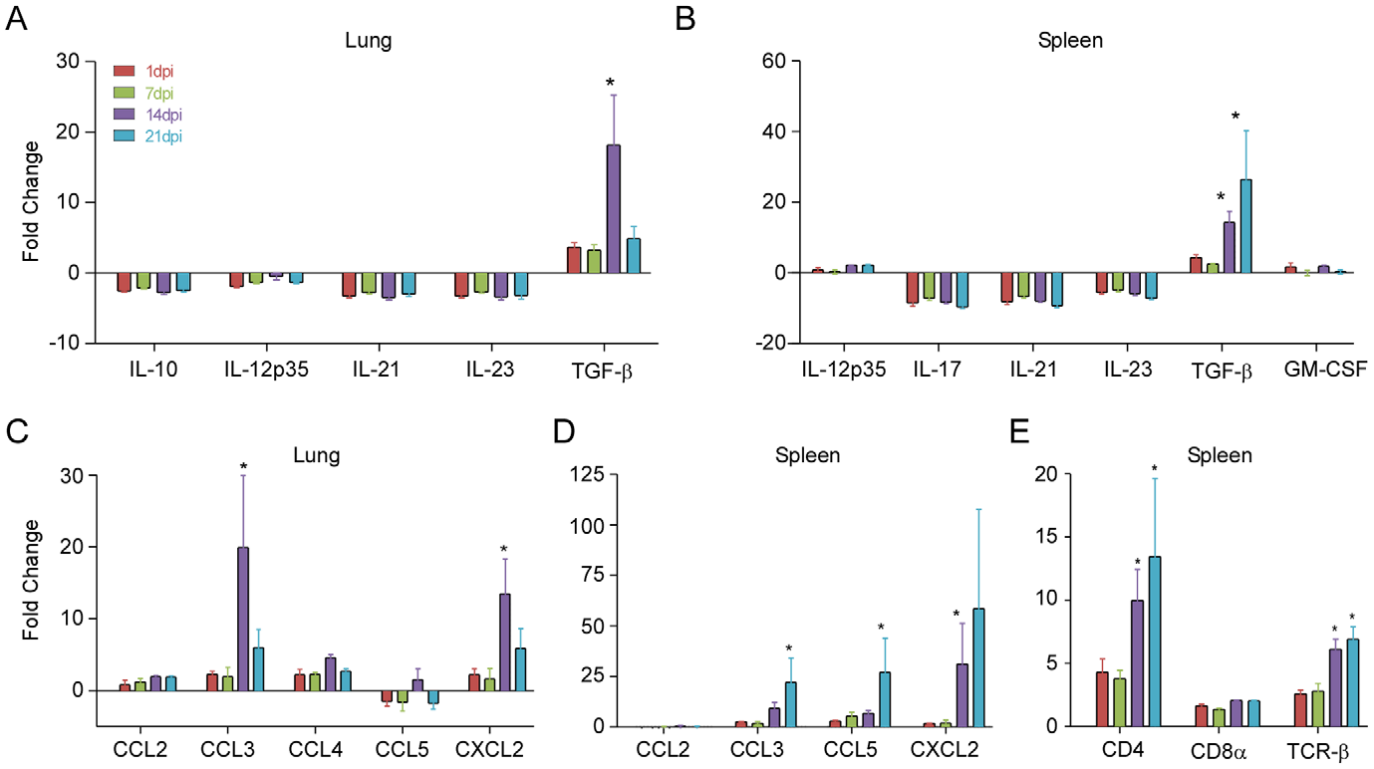
Immune response genes are up-regulated in deer mice following ANDV infection. Expression of pro- and anti-inflammatory cytokine (A & B) and chemokine (C & D), and T-cell marker mRNAs (E) were assessed by real-time RT-PCR in lungs and/or spleens of deer mice inoculated with ANDV. Mice were euthanized and tissues were harvested 1, 7, 14, or 21 dpi. Error bars represent the SEM. A 1-way ANOVA with a Dunnett post test was used to compare the individual groups to control deer mice (* = p<0.05).

Cytokine mRNAs were differentially regulated in the lungs and spleens of inoculated animals (Figure 4A and B). IL-12p35 expression was down-regulated in the lungs at all time points. However, IL-12p35 expression was minimally elevated in spleens at all time points post inoculation. In both the lungs and spleens, increases in TGF-β expression were measured as early as 1 dpi and peak expression was observed in the lungs at 14 dpi. mRNA levels in the spleens of most deer mice peaked at 14 dpi. The expression of anti-inflammatory cytokine mRNAs was slightly down-regulated in the spleens. IL-10, known to inhibit a broad spectrum of activated macrophage/monocyte functions [36], was down-regulated in the lungs at all time points post inoculation. Expression of IL-17, IL-21 and IL-23, cytokines involved in delayed-type responses, were down-regulated in both the spleens and the lungs. IL-21 and IL-23 were down-regulated all time points post inoculation in the lungs.

There was an increase in pro-inflammatory chemokine expression in the lungs and spleens of ANDV-infected deer mice. CCL3 and CCL4, which are CCR5 ligands with antiviral properties and are involved in inflammation [37], [38], [39], [40], were elevated in both spleens and lungs (Figure 4C and D). CCL5 was also elevated in the spleens, but repressed in the lungs at most time points. In addition, CXCL2, which also promotes inflammation, was elevated in both the spleens and the lungs. CCL3, CCL5, and CXCL2 expression in the spleens all peaked at 21 dpi. CCL2, which is pro-inflammatory, but in contrast to CCL3, promotes Th2 immunity, was found to be comparable to the control group at all time points post inoculation.

In spleens, CD4, CD8α, and TCR-β expression was elevated as early as 1 dpi (Figure 4E). Dramatic increases in CD4 expression were seen as early as 1 dpi. CD4 expression was elevated 8-fold in one individual at 1 dpi compared to control animals, and was 44-fold higher than controls in one individual at 21 dpi. CD8α expression was also elevated at 1 dpi, and consistently found at elevated levels in individuals 7, 14, and 21 dpi. TCR-β expression was elevated on 7, 14, and 21 dpi, respectively.

## Discussion

It is was previously unknown whether hantaviruses, which are associated with specific rodent or insectivore host species, could infect, or cause persistent infection in closely-related animal species that are hosts to a related hantavirus. To address this, we inoculated deer mice, the reservoir host of SNV, with ANDV, a South American HCPS-causing hantavirus. Herein, we demonstrate that ANDV infects and replicates in deer mouse embryonic fibroblasts *in vitro*, as well as in deer mice *in vivo*, though it does not cause persistent infection. Infection *in vivo* results in the activation of genes involved in the immune response and clearance of the virus.

New World hantaviruses replicate efficiently in endothelial cells of the microvasculature of rodent hosts [2]. Heart and lung tissues have been described as primary sites for SNV replication in deer mice [27]. Pathogenic hantaviruses use β3 integrin (CD61) for entry into cells, and both SNV and ANDV, at least in part, use this receptor for entry [41]. Therefore, it is not surprising that ANDV might gain entry into deer mouse cells and initiate replication. *In vitro*, ANDV efficiently replicated in deer mouse cells, demonstrating permissiveness and showing that the innate immune response is unable to control the virus in that setting. Detection of dramatically increasing amounts on viral RNA in the supernatants of infected cells suggests that these cells were productively infected by ANDV.

In this study, we used male deer mice as SNV replicates more efficiently in males than females (unpublished observations of BH). Additionally, in Lewis rats experimentally infected with Seoul hantavirus, males had a higher viral load in the lungs than did females [42]. *In vivo*, viral RNA was detected in blood, spleen, lung, and heart tissues of ANDV-inoculated deer mice. While viremia and the presence of viral RNA was detected in these tissues, viral RNA copy numbers were much lower (3–6 logs) at similar time points than those we previously reported in deer mice infected with SNV [25]. It is possible that the ANDV RNA measured in the various tissues was derived from contaminating blood, as the levels of viral RNA in the blood far exceeded that measured in the individual tissues. Likewise, we were unable to identify immunopositive cells by IHC in tissues. However, the time points at which viral RNA was detected differed between the tissues sampled and were consistent within the groups, suggesting that low levels of viral replication and/or transcription probably occurred in these tissues. For example, viral RNA was detected at 14 dpi in the lungs of most animals, but viral RNA was not detected in the hearts until 21 dpi in most deer mice. We used qRT-PCR as a surrogate for viral replication and infection due to its sensitivity and the difficulties associated with isolating hantaviruses in tissue culture. Although we are confident that replication of the viral genome took place within tissues, we did not directly measure a productive infection by virus isolation. Transient and low levels of viral RNA, as an indication of low viral load and replication, could explain the lack of immunohistochemical staining of N antigen in the tissue samples. While IHC has been shown to be a sensitive method to detect hantavirus antigens in human cases [43], viral loads in human clinical disease are often high, and viral load has been directly correlated with disease severity [44], [45]. Similar to other virus infections, low viral load in ANDV-inoculated deer mice, and dispersed virus within the tissues affects sensitivity, and challenges the limits of detection of IHC staining techniques [46].

ANDV elicited high titers of anti-N antibodies in all animals inoculated, and neutralizing antibodies were detected in all animals at 21 and 56 dpi. The development of a neutralizing antibody response might relate to the increase in presumed CD4^+^ T cell expression (measured by elevated CD4 mRNA levels in the spleens), as these cells are necessary for the development of antibody responses. In SNV-infected deer mice, CD4 RNA expression was only moderately elevated at 7 dpi and quickly returned to background levels [25]. Heterologous reservoir rodent species may efficiently mount a neutralizing antibody response that results in limiting virus replication at early time points, whereas the natural rodent hosts may have adapted to hantavirus infection in a way in which the humoral response is delayed, thus allowing for the establishment of a persistent infection.

Evasion of host immune responses is a viral-mediated mechanism to establish persistent infection, and evidence of innate immune evasion has been shown for some hantaviruses [47], [48], [49], [50]. Inoculation of deer mice with ANDV resulted in the increased transcription of chemokine genes in the lungs and spleen, with a concurrent down-regulation of anti-inflammatory and delayed-type immune response genes. The high level of gene regulation in response to ANDV in deer mice further supports the hypothesis that early evasion and down-regulation of host responses may contribute to persistent infection in reservoir hosts, as ANDV is not naturally hosted by deer mice and the virus has not evolved mechanisms for persistence in this species. The RNA encoding the largely anti-inflammatory cytokine TGF-β was markedly up-regulated in ANDV-inoculated deer mice. This may explain why infection was not associated with any clinical signs or histopathological changes, as TGF-β predominantly inhibits inflammatory responses.

Previous studies reporting infection of deer mice with SNV were too different in methods, timing, and nature of the analytes examined to allow a close direct comparison with the present study [25]. However, several aspects of the course of infection and resultant immune responses differed from our previous study where we examined the replication kinetics and immune responses to SNV in deer mice [25]. In our previous study, despite much higher viral replication measured in the lung, SNV infection resulted in a comparatively low level of up-regulation of genes involved in the immune response, where most genes examined were regulated 3-fold or less [25]. In contrast, and despite far less viral replication, ANDV infection resulted in up-regulation of between 5- and 50-fold for several genes. Expression of RNAs for T cell markers in the spleens was higher in deer mice infected with ANDV than in deer mice infected with SNV, in which a predominant CD8α rather than CD4 response occurred [25]. ANDV infection resulted in a higher expression of CD4 and TCR-β RNAs, and comparable expression of CD8α, suggesting that a robust CD4^+^ T cell response may contribute to viral clearance. The immune response to ANDV was also characterized by robust expression of RNAs for Ccr5 ligands (*Ccl3*, *Ccl4*, and *Ccl5*). Enhanced signaling of CD4^+^ T cells can occur through Ccr5 [51], [52]. These genes were only slightly up-regulated upon SNV infection [25].

These data largely support the hypothesis that hantavirus replication and persistence are restricted to the individual reservoirs in which they have adapted, and demonstrates that geographically separated hantaviruses may not be competent for persistent infection of a heterologous host without deliberate attempts at adaptation. Although viral RNA was only detected a very low levels, the production of high titer antibodies and the presence of neutralizing antibodies at later time points, along with the differential regulation of immune-related genes, suggests that inoculation of deer mice with ANDV resulted in a productive infection, and not simply an abortive one. ANDV infection in deer mice provides a unique model of infection resulting in a protective immune response with an absence of virus-associated pathology, and the differences in the responses of the two hosts can be exploited to better understand what determinants favor clearance or persistence. We show that deer mice control ANDV infection with resultant low viral RNA loads. This work serves as the first study to characterize infection of a geographically distinct hantavirus in a heterologous rodent reservoir host resulting in the absence of persistent infection and, importantly, serves to contribute to the body of information regarding what constitutes an effective immune response to infection and what characteristics are associated with the ability to persistently infect a host. Future studies should define the immunological mechanisms that lead to persistence or clearance. Attempts to serially passage ANDV in deer mice to develop a persistence model might lead to insights about what viral genetic changes are needed to develop persistent infection and reduce immunologic recognition of the virus.
